# Chemometric Comparison of High-Pressure Processing and Thermal Pasteurization: The Nutritive, Sensory, and Microbial Quality of Smoothies

**DOI:** 10.3390/foods10061167

**Published:** 2021-05-23

**Authors:** Marko Škegro, Predrag Putnik, Danijela Bursać Kovačević, Ana Petra Kovač, Lidija Salkić, Iva Čanak, Jadranka Frece, Sandra Zavadlav, Damir Ježek

**Affiliations:** 1 Department of Process Engineering, Faculty of Food Technology and Biotechnology, University of Zagreb, Pierottijeva 6, 10000 Zagreb, Croatia; mskegro@pbf.hr (M.Š.); djezek@pbf.hr (D.J.); 2 Department of Food Technology, University North, Trg dr. Žarka Dolinara 1, 48000 Koprivnica, Croatia; pputnik@alumni.uconn.edu; 3 Department of Food Engineering, Faculty of Food Technology and Biotechnology, University of Zagreb, Pierottijeva 6, 10000 Zagreb, Croatia; ana-petra_267@hotmail.com (A.P.K.); lidija.salkic@pbf.hr (L.S.); 4 Department of Biochemical Engineering, Faculty of Food Technology and Biotechnology, University of Zagreb, Pierottijeva 6, 10000 Zagreb, Croatia; iva.canak@pbf.unizg.hr (I.Č.); jfrece@pbf.hr (J.F.); 5 Department of Food Technology, Karlovac University of Applied Sciences, Trg J. J. Strossmayera 9, 47000 Karlovac, Croatia; sandra.zavadlav@vuka.hr

**Keywords:** high-pressure processing, thermal pasteurization, smoothie, storage, quality, bioactive compounds, chemometrics

## Abstract

This study investigated the status of bioactive compounds (phenolic compounds, carotenoids, and vitamin C), changes in color performance, and microbiological quality in smoothies preserved by high-pressure processing (HP) and thermal pasteurization (P) during cold storage at 4 °C for 21 days. Chemometric tools were used to select relevant variables that represent the most useful information for the fast and accurate quality assessment of smoothies. HP was performed at 350 and 450 MPa for 5 and 15 min at room temperature, respectively, while P was performed at 85 °C for 7 min. Smoothies were prepared by blending juices of apple (50%, *v*/*v*), carrot (20%, *v*/*v*), chokeberry (5%, *v*/*v*), Indian banana puree (10%, *w*/*v*), and almond drink (15%, *v*/*v*). The results obtained indicated that lower pressures with a shorter duration of HP showed higher levels of bioactive compounds in the smoothies, compared to the control samples. Compared to P, the HP samples exhibited a greater stability of bioactive compounds during shelf life. HP was found to be highly effective in reducing the native microflora of the smoothies, without subsequent microbial activation during storage. This study demonstrated the usefulness of the chemometric approach in interpreting complex datasets for the effective quality assessment of smoothies treated with different preservation technologies.

## 1. Introduction

Smoothies are considered a typical example of a “superfood”, which is defined as a natural food that could have beneficial effects on human health due to its nutrient composition [1]. They usually consist of mixed fruits/vegetables with added milk, either animal or plant-based. Other studies confirm an association between a high intake of fruits and vegetables and the prevention of chronic diseases, obesity, diabetes, and cardiovascular diseases [2,3]. Smoothies also represent an excellent matrix for the addition of various functional ingredients, such as probiotics or plant extracts, thus offering great potential for the production of functional foods [4,5]. In line with this, smoothies also seem to play a positive role in nutrition, as they are potentially beneficial to health [6,7,8] and can therefore provide increased fruit and vegetable consumption, especially in young age groups [9].

Smoothies are usually prepared fresh without any preservation and therefore have a very short shelf life. Juices and drinks are usually preserved by thermal pasteurization at 84–88 °C for 15–45 min [10]. The main objective of pasteurization is to deactivate thermolabile microorganisms responsible for food spoilage or food poisoning, such as yeasts, molds, and vegetative bacteria [11,12]. In addition to microbial inactivation, thermal pasteurization has also been successfully used to deactivate juice enzymes whose activities can cause oxidative changes during processing and storage [13]. Therefore, thermally treated juices have an extended shelf life of up to several months at refrigerated or room temperature, without a significant loss of quality [14]. However, the application of heat treatment in smoothies can lead to undesirable changes in the functional properties of thermally unstable bioactive compounds (BACs), as well as sensory properties, such as color, taste, and odor [12,15]. Therefore, the application of innovative non-thermal technologies, such as high-pressure processing (HP), has been increasingly explored as a substitute for heat treatment in the processing of fruit and vegetable juices [16].

HP, by applying pressures between 100 and 1000 MPa for several seconds or minutes, effectively deactivates microbial growth and denatures enzymes, with minimal changes in the nutritional and sensory quality [17]. However, the efficiency of this technology is strongly influenced by the operating conditions, such as the pressure level, temperature, and time, followed by the water activity, microbial species, and cell growth phase [18,19]. HP has been approved by the U.S. Food and Drug Administration (FDA) and the U.S. Department of Agriculture (USDA) as a complementary non-thermal pasteurization technique that can ensure microbial safety and significantly extend the shelf life of processed foods [20]. Since smoothies are multi-component systems consisting of various biological macromolecules and BACs that promote microbial growth, the use of HP to extend shelf life and maintain fresh-like quality characteristics can be a major challenge for the food industry.

When consumers consider processed foods (e.g., smoothies), they try to choose products with a significant nutritional value, but also with attractive colors [21]. In contrast to thermal treatment, HP was found to keep the visual color much closer to that of the untreated smoothie [21]. While thermal pasteurization (P) could negatively affect the stability of the BACs in smoothies [22], a recent study showed that HP could even increase the baseline levels of nutritional quality in vegetable smoothies during cold storage [23]. Moreover, HP was found to be a less destructive treatment in terms of the vitamin C stability, compared to thermal processing, which could reduce vitamin C content by 35–44% [24]. In addition, cold storage can negatively affect ascorbic acid content in smoothies over storage time [25], but these changes were less pronounced, compared to thermally treated samples [21].

Some studies suggest that the HP operating conditions (e.g., pressure level, processing time, and processing temperature) may also significantly affect the stability of the BACs in smoothies [24]. Phenolic compounds were 15% higher in smoothies treated below 450 MPa than in those treated at 600 MPa. The authors explained that the longer time to reach final pressurization (400 MPa vs. 600 MPa) may cause this effect [24]. Therefore, the HP processing conditions should be carefully considered to find an optimal HP regime that leads to satisfactory results.

While smoothies are very popular among consumers, many cannot consume these products based on milk of animal origin due to lactose intolerance, allergies, or a trend towards vegetarian diets, so there is a need for more research on the use of non-dairy substitutes in fruit and vegetable smoothies [26]. Consequently, the aim of the present study was to evaluate the use of HP technology (350 and 450 MPa/5 and 15 min/room temperature) and conventional thermal processing (85 °C for 7 min) for almond milk-based smoothies in terms of nutritional value and color parameters during cold storage. A chemometric approach was used to evaluate the changes in the quality parameters of smoothies during storage in relation to the preservation technology applied.

## 2. Materials and Methods

### 2.1. Sample Preparation

Smoothies were prepared by mixing apple juice (50%, *v*/*v*), carrot juice (20%, *v*/*v*), chokeberry juice (5%, *v*/*v*), almond drink (15%, *v*/*v*), and Indian banana puree (10%, *w*/*v*). Apple (cv. Golden Delicious) and carrot juices were prepared from raw materials purchased from a local market. A cold-pressed juicer (300 W TEFAL Infinity Press Revolution ZC500H38, France), with a speed of 80 rpm and a filter diameter of 0.3 mm, was used to prepare cloudy apple juice, while a juicer (1000 W BOSCH MES 4000, Germany), with a filter diameter of 0.5 mm, was used to prepare carrot juice. Approximately 8.5 L of apple juice and 3.4 L of carrot juice were prepared, which required around 14 kg of apples and 7 kg of carrots. The chokeberry juice (cca 1 L) and Indian banana puree (cca 1.7 kg) were kept frozen and thawed at room temperature before the smoothie preparation. All ingredients were homogenized using a hand blender (170 W SIEMENS MQ 33001, Germany) and then filled into polyethylene terephthalate (PET) bottles, which were then subjected to a high pressure (HP). Two bottles of 200 mL for one HP and one storage time were filled with a smoothie, resulting in a total of 16 HP-bottles. For the pasteurization, three glass bottles (500 mL) were filled with the smoothies for each storage regime, i.e., a total of 4 P-bottles. Two glass bottles (500 mL) were filled with the control samples (untreated) and represented fresh smoothies.

### 2.2. High-Pressure Processing

The prepared smoothies were treated with a high pressure using an HP device from Stansted Fluid Power (UK). The samples in plastic bottles were previously vacuumed in the disposable plastic bags provided in a vacuum device (STATUS SV2000) and then placed in a high-pressure container filled with a pressurized liquid (propylene/glycol: water in a 50:50 ratio). The samples were subjected to a pressure of 350 MPa and 450 MPa for 5 and 15 min, respectively, at room temperature (≈20 °C), according to the experimental design given in Table 1.

### 2.3. Thermal Pasteurization (P)

The thermal pasteurization (P) regime was selected from the literature for the smoothie with a similar composition [27]. A batch laboratory pasteurizer (PS-100, Oprema Ludbreg d.o.o. Ludbreg, Croatia) was used for the P of smoothies at 85 °C for 7 min. Preliminary studies showed that the pasteurizer needs 19 min to reach the set temperature, so the total pasteurization time of the smoothie samples was 26 min. Pasteurized smoothies were subjected to standard storage regimes and used for analysis at the defined storage times (Table 1).

### 2.4. Determination of Bioactive Compounds

All absorbance measurements were conducted with a UV/Vis spectrophotometer (VWR UV-1600PC Spectrophotometer, VWR International, Pennsylvania, PA, USA). For each sample duplicate measurements were performed.

#### 2.4.1. Ultrasound-Assisted Extraction of Bioactive Compounds

The extraction was carried out in the ultrasonic processor, Bandelin Sonorex (Germany), operating at a 40 kHz frequency, with modified methods from previously published data [28,29]. Briefly, 5 g (±0.0001) of a smoothie, along with 20 mL of ethanol (96%), as an extraction solvent, was mixed in an Erlenmeyer flask, and the mixture was sonicated for 15 min at T = 50 °C. Afterwards, the extract was filtered through Whatman filter paper No. 40 (Whatman International Ltd., Kent, UK) and made up to 25 mL in a volumetric flask with extraction solvent. All extracts were prepared in duplicates. Prior to analysis, the extracts were stored at T = 4 °C in an inert gas atmosphere.

#### 2.4.2. Determination of the Total Phenolic Content (TPC)

The total phenolic contents were determined according to a modified method from the literature [19]. The reaction mixture contained: 0.4 mL of extract, 0.4 mL of Folin-Ciocalteu reagent, and 4 mL of sodium carbonate solution (75 g L^−1^). After 1 h of incubation at room temperature in the dark, the absorbance was measured at 725 nm using a spectrophotometer. A blank sample was prepared with distilled water, instead of extract. A calibration curve was prepared using a standard solution of gallic acid (10–250 mg L^−1^), and the results were expressed as mg of gallic acid equivalent (GAE) per 100 mL of the sample.

#### 2.4.3. Determination of the Total Flavonoids (TFL)

The total flavonoids were determined according to Chang et al. [30]. First, 0.5 mL of extract was mixed with 1.5 mL of 96% ethanol, 0.1 mL of 10% Al(NO_3_)_3_, 0.1 mL of 1 M potassium acetate, and 2.8 mL of distilled water. After 30 min of incubation at room temperature, the absorbance of the reaction mixture was measured at 415 nm using a spectrophotometer. A calibration curve was prepared using a standard solution of quercetin (10–100 mg L^−1^), and the results were expressed as mg of quercetin equivalent (QE) per 100 mL of the sample.

#### 2.4.4. Determination of the Total Hydroxycinnamic Acids (HCA)

To determine the content of total hydroxycinnamic acids, the procedure proposed by Howard et al. [31] was applied, with slight modifications. In a test tube, 0.25 mL of extract was mixed with 0.25 mL of 1 g L^−1^ HCl (in 96% ethanol) and 4.55 mL of 2 g L^−1^ HCl (in distilled water) and stirred in a vortex for 10 s, then allowed to react in the dark for 30 min at room temperature. After this time, the solution absorbance was measured at 320 nm in a spectrophotometer. As a blank sample extraction, solvent, instead of extract, was used. A standard solution of chlorogenic acid (10–100 mg L^−1^) was used for the calibration curve preparation, and the results were expressed as mg of chlorogenic acid equivalent (CAE) per 100 mL of the sample.

#### 2.4.5. Determination of the Total Carotenoids (CAR)

The total carotenoids were determined using the method of Lichtenthaler and Buschmann [32]. The absorbance maxima of the smoothie extracts in contrast to the blank (96% ethanol) were read at 470 nm and 649 nm for Chl a and at 664 nm for Chl b using the UV/Vis spectrophotometer, and the total carotenoids C(x + c) [xanthophylls and carotenes] were calculated from the following equations:

Chl a (µg mL^−1^) = 13.36 A664 − 5.19 (1)

Chl b (µg mL^−1^) = 27.43 A649 − 8.12 (2)

C(x + c) (µg mL^−1^) = (1000 A470 − 2.13 Chl a − 97.63 Chl b)/209 (3)

The results were expressed as mg per 100 mL of the sample.

#### 2.4.6. HPLC-DAD Determination of Vitamin C (Ascorbic Acid)

Separations and quantifications of vitamin C were performed using HPLC equipment (Thermo Scientific Accela HPLC system, Thermo Fisher Scientific, Waltham, MA, USA) on Nucleosil 100-5C18, 5 µm (250 mm × 4.6 mm I.D.) column (Phenomenex, Los Angeles, CA, USA). The separation was performed using a standard method (HRN EN 14130:2005—Determination of vitamin C by HPLC). The LOD (limit of detection) and LOQ (limit of quantitation) values were as follows: 0.2 mg and 100 mL^−1^ and 1.6 mg and 100 mL^−1^, respectively. The content of vitamin C was expressed in mg of ascorbic acid (the sum of ascorbic acid and its oxidative form of dehydroascorbic acid) per 100 mL of the sample.

### 2.5. Instrumental Color Measurement

Color measurements were made using diffuse reflectance spectrophotometry on a colorimeter (CM-3500d, Konica-Minolta, Tokyo, Japan). A pulsed xenon lamp was used for the standard D 65 illumination. All necessary measurement settings were made using the Spectramagic NX program (Konica-Minolta, Tokyo, Japan). The geometry d/8 was chosen, where the surface of the sample is viewed at an angle of 8° to its normal. L* (lightness), a* (green to red), and b* (yellow to blue) were measured, and the values of C (chroma), H (hue), and ∆E_ab_ (colour difference) were calculated (1, 2, 3). The values obtained represent the average of 4 replicates.

(4) $$C^{*}=\sqrt{a^{*2}+b^{*2}}$$

(5) $$H^{*}=arctan\frac{b^{*}}{a^{*}}$$

(6) $$\Delta E_{ab}=\sqrt{{\left(L_{2}-L_{1}\right)}^{2}+{\left(a_{2}-a_{1}\right)}^{2}+{\left(b_{2}-b_{1}\right)}^{2}}$$

L_1_, a_1_, and b_1_ are the color parameters for the control samples, and L_2_, a_2_, and b_2_ are the color parameters of the treated samples.

### 2.6. Microbial Analyses

Classical microbiological methods were used to monitor the microbiological quality of the smoothies stored for 21 days at 4 °C. The tested microorganisms were selected according to the prescribed regulations for the microbiological criteria for foodstuffs (EC, 2073/2005) [33].

A volume of 1 mL of each sample was homogenized in 9 mL of sterile water and serially diluted before plating (pour plate method for total aerobic mesophilic bacteria and spread plate method for other bacteria) on selective media. All analyses were made in triplicates. The total aerobic mesophilic bacteria were counted after incubation on nutrient agar (Merck, Darmstadt, Germany) at 37 °C for 48 h, *Enterobacteriaceae* after incubation on Violet Red Bile Glucose (VRBG) agar (Biolife, Milan, Italy) at 37 °C for 48 h, and mold and yeasts after incubation on potato dextrose agar (Biolife) at 25 °C for 96 h. *Salmonella* sp. was grown in a Rappaport-Vassiliadis (RV) Salmonella enrichment broth (Merck), followed by subculturing on xylose lysine deoxycholate (XLD) agar (Biolife) at 37 °C for 24–48 h. *Listeria monocytogenes* was detected using a two-step selective enrichment procedure in Fraser broth, followed by subculturing on PALCAM agar (Merck) at 37 °C for 24 h. The microbial growth was determined using traditional plate counting, and the results were expressed as the colony forming units per milliliter of juice (CFU mL^−1^).

### 2.7. Statistical Analysis

Descriptive statistics were used for the characterization of the sample. Discrete variables and factor scores were tested by MANOVA. Exploratory hierarchical Ward’s cluster analysis was used for measuring standardized similarities in samples. For nonparametric analysis, a Kruskal Wallis test was employed. In order to check the structure of specific variables, factor analysis (Principal Component Analysis; PCA) was performed on selected variables to estimate the overall changes in the nutritive value of samples for various combinations of independent variables. The appropriateness of factor analysis was tested by a Kaiser-Mayer-Olkin test (KMO) and Bartlett’s Test of Sphericity. The factor analysis score was calculated by the linear regression method. The level of significance for all tests was α ≤ 0.05, and the results were analyzed using SPSS software (v.22).

## 3. Results and Discussion

### 3.1. Application of Chemometrics for the Evaluation of Processing and Food Quality of Smoothies

#### 3.1.1. Thermal Pasteurization and High-pressure Processing

**Figure 1:**
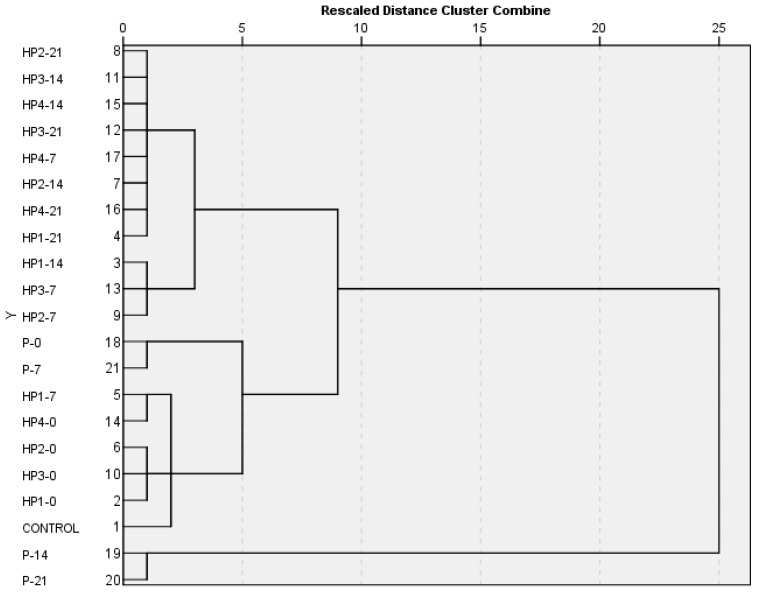
Results of the hierarchal cluster analysis of averaged and standardized samples.

The first step in data analysis is to explore the relationships within the dataset. Exploratory hierarchical Ward’s cluster analysis revealed that when all samples were observed on standardized similarities (L*, a*, b*, C*, H*, and total phenolic content (TPC, mg 100 mL^−1^), total hydroxycinnamic acid content (HCA, mg 100 mL^−1^), total flavonoid content (TFL, mg 100 mL^−1^), vitamin C content (mg 100 mL^−1^), and total carotenoid content (CAR, mg 100 mL^−1^), the samples most similar to the controls (untreated) were the HP1-0, HP2-0, HP3-0, HP4-0, and HP1-7. The next most similar characteristics were noticed for a P-0 and P-7. From the dendrogram, it can be concluded that for most of the storage, the high-pressure processing samples were more similar to the control samples than the thermally pasteurized (P) samples (Figure 1). This was even more obvious with the passing of storage, as the pasteurized samples at 7th and 21st day formed separate and distinct clusters from the control samples. This implied a positive influence of nonthermal HP on food quality over thermal pasteurization.

Furthermore, nonparametric analysis revealed that HP samples had similar characteristics as untreated samples for L*, b*, C*, H*, TFL (mg 100 mL^−1^), and CAR (mg 100 mL^−1^) contents. TPC (mg 100 mL^−1^), HCA (mg 100 mL^−1^), vitamin C (mg 100 mL^−1^), and CIEL a* were found in lower amounts in the HP samples (Table 2) than in the untreated samples. The average values for the control sample were: L* = 45.06 ± 0.01; a* = 16.70 ± 0.02; b* = 17.82 ± 0.01; C* = 24.42 ± 0.01; H* = 46.87 ± 0.05; TPC = 782.57 ± 20.00; HCA = 51.51 ± 2.60; TFL = 58.58 ± 0.64; vitamin C = 27.48 ± 0.13; and CAR = 14.97 ± 0.85.

Nonparametric analysis (Table 3) revealed that the HP samples had similar characteristics as the thermally pasteurized samples for L*, a*, H*, HCA (mg 100 mL^−1^), and CAR (mg 100 mL^−1^). TPC (mg 100 mL^−1^), TFL (mg 100 mL^−1^), b*, and C* were higher for the pasteurized, while the HP samples had a higher vitamin C content (mg 100 mL^−1^). The average values for thermal pasteurization were: L* = 36.88 ± 11.02; a* = 17.67 ± 3.99; b* = 24.47 ± 6.74; C* = 30.19 ± 7.79; H* = 26.78 ± 27.52; TPC = 857.99 ± 65.34; HCA = 36.76 ± 9.03; TFL = 58.18 ± 5.29; vitamin C = 2.00 ± 2.45; and CAR = 12.49 ± 1.94.

The results obtained from the nonparametric analysis (Table 4) revealed that the control samples had similar studied parameters as the thermally pasteurized samples for all bioactives, except for HCA and vitamin C. Both of these bioactive compounds were higher in the control samples.

To consider the effect of pasteurization on the bioactive compounds in the smoothies, it can be observed that the TPC was lower in the HP-treated than in the pasteurized samples (685.93 ± 2.89 vs. 857.99 ± 5.15 mg 100 mL^−1^). It seems that thermal treatment causes the release of phenolic compounds from the fruit matrix, resulting in the higher TPC content in the thermally treated samples [34]. Moreover, the pasteurized samples seem to show more TFL, compared with what would be found in the HP samples (58.18 ± 0.34 vs. 47.62 ± 0.46 mg 100 mL^−1^). This increased stability of TFL might be related to the release of monomers and dimers during the thermally induced hydrolysis of heat-labile phenolic compounds [35]. HCA showed a lower stability during the thermal treatment than the control samples did. However, there were no significant differences in the HCA concentration between the HP and pasteurized samples.

The vitamin C content in the control samples seemed to be reduced by pasteurization, a behavior also shown by other researchers in the thermal treatment of various smoothies and juices [12]. Similar to our results, Keenan et al. [24] reported that HP provided a better vitamin C retention, compared to heat-treated smoothies. Moreover, the vitamin C content of strawberry puree after HP (300 and 500 MPa/1, 5 or 15 min/0 °C) was significantly higher than after thermal pasteurization (90 °C/15 min) [36]. The retention of ascorbic acid was slightly higher in orange juice and milk drink at 400 MPa/15 °C/5 min than that heated at 90 °C/15 s [37]. The vitamin C loss of the pasteurized samples could be due to the thermal degradation of vitamin C during pasteurization, which could be avoided by an adequate selection of the pulp [26]. Moreover, vitamin C is a thermolabile compound and, as such, undergoes enzymatic and chemical oxidation during processing. Oxidative enzymes and vitamin C may come into contact when the food matrix is disrupted by thermal pasteurization, while HP is able to inactivate these enzymes [38,39,40].

When considering the thermal stability of CAR, it can be seen that pasteurization did not alter these compounds, compared with the control samples. This was not surprising, as a recent study showed that mild (90 °C/20 s) and intense (120 °C/20 s) heat treatments were able even to improve the stability of carotenoids through release and micellarization in carrot juice-papaya-mango and carrot juice-pumpkin-mango smoothies [41]. In general, it can be concluded that the CAR were stable pigments, regardless of whether thermal pasteurization or HP was used.

#### 3.1.2. Nutritive Index as a Measure of the Nutritive Quality of Smoothies

To cumulatively observe the changes in nutritive value, PCA factor analysis was used to create a single factor, which was labeled “Nutritive index” and tested against processing parameters for HP. This explained 75% of the total variance (Table 5). The crystalized factor included the contents of hydroxycinnamic acid, vitamin C, and total carotenoids. KMO = 0.71, which ensured the sampling adequacy, while Bartlett’s Test of Sphericity = 48.15 at *p* ≤ 0.01. A higher value of the Nutritive index simultaneously implied higher values for HCA, vitamin C, and CAR (Table 6).

### 3.2. The Changes of Bioactive Compounds in Smoothies under High Pressure, Pasteurization, and Storage

Since smoothies were prepared from different plant materials (e.g., fruits, vegetables, and almond milk), the effects of HP and P on different bioactive compounds were observed to clearly investigate their stability in such heterogeneous systems. The changes in the total content of phenols, hydroxycinnamic acids, flavonoids, vitamin C, carotenoids, and overall nutritive quality of the pressurized and pasteurized smoothies are shown in Table 7 and Table 8.

#### 3.2.1. The Influence of High-pressure Processing Parameters on the Nutritive Value of Smoothies

Compared to the untreated samples, HP decreased TPC by 12%, with no significant effect of the pressure. The decrease in TPC after HP could be explained by the high residual activity of enzymes (e.g., peroxidase and polyphenol oxidase) responsible for catalyzing the oxidation of phenols [42]. The decrease in TPC can also be attributed to the polymerization of phenolic compounds with proteins [43]. In contrast, Barba et al. [35] found a higher TPC in HP-treated orange juice-milk beverages, compared to control samples, without a significant influence of pressure (300 MPa and 400 MPa). In another smoothie formulation (orange juice: 59%, apples: 15%, carrots: 15%, beet 80 leaves: 6% and beet stalks: 5%), treated under 630 MPa/6 min, no changes in TPC were observed, compared to control samples [44]. Smoothies are complex food matrices; therefore, the effect of a high pressure on the stability of TPC may be different for different food matrices [45], so this should be tested prior to mass manufacturing. Moreover, the prolongation of HP decreased TPC, which is in agreement with previous literature reports [43].

When the other phenolic compounds were considered, the HP time and pressure had no significant effects on the HCA content. In contrast, Baron et al. [46] showed a significant increase in the HCA in apple juice treated with HP (200–600 MPa/15–65 °C/2–9 min). The authors explained the increase in the HCA content upon HP by the demonstrated release of phenolic compounds from the food matrix caused by the deleterious effects of HP on cell structure. In addition, the increased pressure decreased the content of TFL. Kaşıkcı and Bağdatlıoğlu [47] reported that the TFL of water-fruit juice beverages was decreased, while the TFL of soy milk-fruit juice beverages and milk-fruit juice beverages was increased after HP at 400 MPa/40 °C/5 min. In contrast, a recent study confirmed that HP at 600 MPa decreased the concentration of all flavanols in apples, which are the predominant flavonoid subclass in apples, and the decrease (46–53%) was higher for flavanols with a high molecular weight and larger number of hydroxyl groups [48].

HP at lower pressures and temperatures for a shorter treatment duration could be considered as an effective technology to improve the vitamin C stability in fruit and vegetable products [49]. While considerable levels of vitamin C and total carotenoids were detected, the observed results showed that an increased pressure and longer treatment time decreased the levels of vitamin C and carotenoids in the smoothie samples. Similarly, prickly pear beverages treated at 550 MPa/≥2 min showed a loss of 3–15% of vitamin C [50]. A greater depletion of vitamin C during HP may be due to oxidation during adiabatic heating or residual enzymatic activity [49].

#### 3.2.2. The Influence of Storage on the Nutritive Value of Smoothies

The effect of storage on HP-treated and pasteurized smoothies on the changes in bioactive compounds was studied during cold storage (4 °C) for 21 days. The TPC of both pasteurized and HP-treated smoothies decreased significantly during storage (Table 6 and Table 7). This behavior was more significant for the pasteurized samples, for which a decrease from 9.83% to 19.51% was observed after 21 days, compared to the control. However, the loss of TPC during storage was less pronounced in the HP samples (3.42–4.59%). The observed results indicated that the TPCs in the HP samples were more stable during storage than in the pasteurized samples. Similar results were previously reported for orange juices treated with HP and P and stored under refrigeration [51].

During the storage of HP samples, the content of TFL increased until day 7, then returned to the baseline values and remained constant until the 21st day. The increase in TFL after the 7th day of storage in the HP samples could be attributed to the presence of residual polyphenol oxidase and peroxidase activity, which can break the bonds of high-molecular-weight phenolic compounds, such as procyanidins, to release particle units and monomeric flavanols [38].

HP is found to affect the alterations in the quaternary, tertiary, and secondary structure of enzymes and could thus modify the activity of enzymes, inducing an activation at lower pressures and inactivation at higher pressures [52]. The TFL in the pasteurized samples remained stable during storage, although it was reduced by 5.49%, compared to the control samples. Similarly, HCA showed less stability after 21 days of storage in the P samples, compared to the HP samples, against controls, with a reduction of 51.85% and 39.68%, respectively.

As expected, vitamin C proved to be the most sensitive bioactive compound during storage. In the P samples, the vitamin C content steadily decreased during the study and was completely lost by the 14th day of storage. When the HP juices were stored, it was found that, after 7 days of storage, the vitamin C content was reduced by 50%.

Storage reduces the CAR content in the HP and P smoothies by 30.65% and 30.11%, respectively. While the increase in temperature during thermal treatment could induce the dissolution of carotenoids, followed by their release into the solution after the thermal degradation of the cell structure, which could be the explanation for the significantly higher CAR content of the pasteurized sample [53], the obtained results showed that a higher CAR content was observed in HP samples (13.30 mg 100 mL^−1^), as compared to P samples (12.59 mg 100 mL^−1^).

Judging from the Nutritive index, lower pressures yielded a higher nutritional value in the samples. Similarly, the samples treated with HP for shorter times had a higher nutritional value. A steady loss of nutritional value in the samples was evident with prolonged storage. Despite the degradation caused by the HP, the treated smoothies can be considered as rich sources of various bioactive compounds, even during the storage period.

### 3.3. Color Changes of Smoothies during Storage for High-Pressure and Pasteurization Samples

The retention of color parameters in thermally or nonthermally treated smoothies during cold storage is another relevant aspect of fruit processing quality. There are different literature reports on whether HP or P provides a higher color retention in smoothies. The changes in the CIEL*a*b parameters of untreated and high pressure-treated smoothies and their evaluation during the 21-day storage period are shown in Table 9, while the effect of pasteurization on color values during storage is summarized in Table 10.

As can be seen in Table 8, an increased pressure and longer treatment time causes the samples to become darker, which is consistent with previous reports [54]. A similar but not so linear relationship was observed for the duration of storage. Here, a darkening of the samples is observed after the 7th day of storage. Similarly, milk-based HP smoothies showed a slight gradual decrease in L* values during storage (6% decrease for HP-450 and 7% for HP-600) [54].

When comparing the a* parameter from the untreated smoothie, our results showed a decrease of 10.18% and 14.80% for the smoothies treated below 350 MPa and 450 MPa, respectively. In addition to the increased pressure, the duration of treatment also affected the decrease in the CIE value a*. The loss of red color in the HP samples could be attributed to the residual enzyme activity inducing an enzymatic browning of phenolic compounds [55].

The b* values showed significant changes as a function of pressure, treatment time and storage days. The increased pressure and length of treatment time decreased the b* value. The length of storage had a similar effect on the b* parameter, with b* decreasing after the 7th day of storage. The observed change in yellowing could be due to the residual enzymatic activity (e.g., PPO, POD), associated with enzymatic browning [56].

The increased pressure and treatment time decreased chroma (C*), while storage increased it until the 7th day, then it started to decrease and remained constant until the end of storage. Increasing the pressure and increasing the treatment time decreased the hue value (H*), while increasing the storage increased this colorimetric value. However, it is interesting to note that the TFL during storage follows similar patterns as those observed for the colorimetric values, suggesting that the TFL could be responsible for the colorimetric changes in the samples during storage.

The total color difference (ΔΕ) reflects the extent of the color change between the untreated and treated samples. The differences in perceivable color can be classified analytically as not noticeable (0–0.5), slightly noticeable (0.5–1.5), noticeable (1.5–3.0), very visible (3.0–6.0), and great (6.0–12.0) [57]. On average, all of the samples were in the range of 1.5–3.0, representing a significant difference in color, compared to the control samples. The increased pressurization increased the color change, while the increased treatment time decreased it. The duration of storage was positively related to the increased color change; however, the values increased until the seventh day of storage and decreased thereafter.

Compared with the untreated sample, the L* and b* values of the smoothies were increased by P. However, during the storage period, the L* values were found to decrease, while a* and b* increased, as compared to day 0, indicating that the P smoothies became darker and more intensely reddish and yellowish. The observed color changes could be explained by an increase in non-enzymatic browning due to a Maillard reaction and pigment destruction [58].

When analyzing the H* value, it can be seen that it was affected by thermal treatment and storage time, whereas higher H* values were found in the P, as compared to the control samples, which can be attributed to the oxidation reactions catalyzed by the temperature, resulting in degradation products [22]. In addition, lower H* values were found with the prolonged storage time. Similar to redness and yellowness, the chroma values were significantly higher due to the elongated storage time, which means a higher color intensity of the samples.

The ΔΕ values for all P smoothies increased significantly as the storage time progressed. Therefore, the HP smoothies retained their color better and underwent less color change during storage. The obtained results are in accordance with the results obtained for HP and P mango smoothies during storage, where the P samples generated a greater color difference with respect to fresh juice [59].

### 3.4. Microbial Stability of Smoothies during Storage for High-Pressure and Pasteurization Samples

The microbial counts of the HP, P, and control samples during storage at 4 °C are shown in Table 11. A high number of aerobic mesophilic bacteria, enterobacteria, yeasts, and molds were recorded immediately after the preparation of the control sample. Since the control bottles were bloated due to the high number of yeasts and the contents were leaking, the microbiological analysis of this sample was not continued after the third day. Samples treated with HP (350 MPa/5 min, 350 MPa/15 min, 450 MPa/5 min, and 450 MPa/15 min) or pasteurization showed none of the tested microorganisms throughout the 21 days of storage. These results are in agreement with Andrés et al. [60], who observed a decrease in the number of microorganisms and an extension of shelf life to 45 days after treating milk and soy smoothies with HP or pasteurization. In addition, a recent study investigated the shelf life of fruit and vegetable smoothies treated with HP [44]. The results showed a significant reduction in microbial counts and product stability during the 21-day storage period. Microbial inactivation by HP or pasteurization has been demonstrated in numerous studies with fruit and vegetable smoothies and juices. The effect of these methods causes cell wall breakdown, protein denaturation, and DNA degradation [61].

## 4. Conclusions

The chemometric approach can be useful for future applications in the food industry involving the differentiation of foodstuff, comparison of their classification according to applied technology, and quality prediction, allowing for fast and accurate characterizations of food. In this work, the conventional thermal pasteurization (85 °C/7 min) and high-pressure processing (350 and 450 MPa/5 and 15 min/room temperature) of smoothies during 21 days of cold storage were studied in terms of nutritional and microbial quality. An increased pressure did not affect TPC and HCA but decreased the levels of TFL, CAR, and vitamin C in the smoothie samples. A prolonged treatment time negatively affected the content of TPC, CAR, and vitamin C. In HP-smoothies, TFL showed the best stability, while vitamin C was found to be the most unstable during storage.

All the investigated phenolic compounds were observed in higher concentrations in the P samples, as compared to the HP-ones, while significantly lower contents of vitamin C and CAR were found in the P samples in comparison to the HP samples. The storage of the pasteurized smoothies, however, led to significantly higher losses of all the investigated compounds, compared to the HP samples, indicating that although pasteurization favored a better stability of some bioactive compounds, a greater stability was found for the HP samples during storage.

This research showed that high-pressure processing can be a viable choice for the preservation of smoothies’ quality during storage. This conclusion is based on the data, which strongly show that the high-pressure technology is able either to outperform or achieve the same results as conventional thermal pasteurization.

Since smoothies are complex food matrices, the stability of various bioactive compounds and the quality of the rest of the food should be tested prior to mass manufacturing them. Hence, more research on other successful applications of the technology in this part of food market is recommended.

## Figures and Tables

**Table 1:** Experimental design of the study.

Sample ID	Treatment Type	Shelf-Life Analysis (Days)	High Pressure (MPa)	Treatment Time (Min)
1	Control	0	0	0
2	P-0	0	0	0
3	HP1-0	0	350	5
4	HP2-0	0	350	15
5	HP3-0	0	450	5
6	HP4-0	0	450	15
7	P-7	7	0	0
8	HP1-7	7	350	5
9	HP2-7	7	350	15
10	HP3-7	7	450	5
11	HP4-7	7	450	15
12	P-14	14	0	0
13	HP1-14	14	350	5
14	HP2-14	14	350	15
15	HP3-14	14	450	5
16	HP4-14	14	450	15
17	P-21	21	0	0
18	HP1-21	21	350	5
19	HP2-21	21	350	15
20	HP3-21	21	450	5
21	HP4-21	21	450	15

HP—High Pressure-treated samples; P—pasteurized sample. Three bottles were used for each treatment (P or HP), with a total of 12 bottles for P and 48 bottles for HP.

**Table 2:** Kruskal Wallis test statistics for the HP vs. untreated samples (controls).

	L*	a*	b*	C*	H*	TPC	HCA	TFL	Vitamin C	CAR
Chi-Square	0.000	5.487	0.000	3.086	3.086	4.200	5.486	2.594	5.486	1.206
Df	1	1	1	1	1	1	1	1	1	1
Asymp. Sig.	1.000	0.019	1.000	0.079	0.079	0.040	0.019	0.107	0.019	0.272

L*, a*, b*, C*, H*—color parameters; TPC—total phenolic content; HCA—total hydroxycinnamic acids; TFL—total flavonoids; CAR—total carotenoid content.

**Table 3:** Kruskal Wallis test statistics for the HP vs. thermal pasteurization samples.

	L*	a*	b*	C*	H*	TPC	HCA	TFL	Vitamin C	CAR
Chi-Square	0.371	0.929	8.070	5.603	0.659	17.864	0.370	8.458	17.033	1.245
df	1	1	1	1	1	1	1	1	1	1
Asymp. Sig.	0.543	0.335	0.005	0.018	0.417	0.000	0.543	0.004	0.000	0.264

L*, a*, b*, C*, H*—color parameters; TPC—total phenolic content; HCA—total hydroxycinnamic acids; TFL—total flavonoids; CAR—total carotenoid content.

**Table 4:** Kruskal Wallis test statistics for the untreated vs. thermal pasteurization samples.

	L*	a*	b*	C*	H*	TPC	HCA	TFL	Vitamin C	CAR
Chi-Square	0.000	0.000	1.091	0.000	0.000	3.341	4.364	1.098	4.645	2.455
df	1	1	1	1	1	1	1	1	1	1
Asymp. Sig.	1.000	1.000	0.296	1.000	1.000	0.068	0.037	0.295	0.031	0.117

L*, a*, b*, C*, H*—color parameters; TPC—total phenolic content; HCA—total hydroxycinnamic acids; TFL—total flavonoids; CAR—total carotenoid content.

**Table 5:** Loading of the “Nutritive Index” factor.

	Components
	1
Vitamin C (mg 100 mL^−1^)	0.75
Total carotenoids (mg 100 mL^−1^)	0.81
Hydroxycinnamic acids (mg 100 mL^−1^)	0.70
Explanation of variance	75%
Eigenvalue	2.26

**Table 6:** Pearson’s correlations with the Nutritive index.

	Nutritive Index	Hydroxycinnamic Acids (mg 100 mL^−1^)	Vitamin C (mg 100 mL^−1^)	Total Carotenoids (mg 100 mL^−1^)
Nutritive Index	1	0.80 **	0.89 **	0.95 **
Hydroxycinnamic acids (mg 100 mL^−1^)		1	0.54 **	0.65 **
Vitamin C (mg 100 mL^−1^)			1	0.82 **
Total carotenoids (mg 100 mL^−1^)				1

** Correlation is significant at the 0.01 level (2-tailed).

**Table 7:** Changes in the bioactive compounds and nutritive index in smoothie samples under HP during storage.

Variable	n	TPC	HCA	TFL	Vitamin C	CAR	Nutritive Index
**Pressure**		*p* = 0.48 ^‡^	*p* = 0.42 ^‡^	*p* ≤ 0.01 ^†^	*p* ≤ 0.01 ^†^	*p* ≤ 0.01 ^†^	*p* ≤ 0.01 ^†^
350 MPa	16	688.0 ± 4.1 ^a^	35.7 ± 0.5 ^a^	49.3 ± 0.66 ^a^	11.72 ± 0.1 ^a^	13.8 ± 0.1 ^a^	0.21 ± 0.03 ^a^
450 MPa	16	683.8 ± 4.1 ^a^	35.1 ± 0.5 ^a^	45.9 ± 0.66 ^b^	7.81 ± 0.1 ^b^	12.8 ± 0.1 ^b^	−0.23 ± 0.03 ^b^
**Time**		*p* ≤ 0.01 ^†^	*p* = 0.94 ^‡^	*p* = 0.26 ^‡^	*p* ≤ 0.01 ^†^	*p* ≤ 0.01 ^†^	*p* ≤ 0.01 ^†^
5 min	16	697.7 ± 4.1 ^a^	35.4 ± 0.5 ^a^	48.2 ± 0.66 ^a^	10.41 ± 0.1 ^a^	13.5 ± 0.1 ^a^	0.08 ± 0.03 ^a^
15 min	16	674.1 ± 4.1 ^b^	35.4 ± 0.5 ^a^	47.1 ± 0.66 ^a^	9.12 ± 0.1 ^b^	13.1 ± 0.1 ^b^	−0.09 ± 0.03 ^b^
**Storage**		*p* ≤ 0.01 ^†^	*p* ≤ 0.01 ^†^	*p* ≤ 0.01 ^†^	*p* ≤ 0.01 ^†^	*p* ≤ 0.01 ^†^	*p* ≤ 0.01 ^†^
0 days	8	708.8 ± 5.8 ^a^	39.9 ± 0.7 ^a^	44.3 ± 0.93 ^b^	16.37 ± 0.1 ^a^	16.3 ± 0.1 ^a^	1.18 ± 0.05 ^a^
7 days	8	684.6 ± 5.8 ^b^	37.1 ± 0.7 ^b^	54.2 ± 0.93 ^a^	8.84 ± 0.1 ^b^	13.8 ± 0.1 ^b^	0.13 ± 0.05 ^b^
14 days	8	674.1 ± 5.8 ^b^	33.6 ± 0.7 ^c^	44.9 ± 0.93 ^b^	8.26 ± 0.1 ^c^	11.8 ± 0.1 ^c^	−0.47 ± 0.05 ^c^
21 days	8	676.2 ± 5.8 ^b^	31.1 ± 0.7 ^d^	47.0 ± 0.93 ^b^	5.60 ± 0.1 ^d^	11.3 ± 0.1 ^d^	−0.87 ± 0.05 ^d^
**SAMPLE MEAN**	32	685.9 ± 2.9	35.42 ± 0.4	47.6 ± 0.46	9.8 ± 0.1	13.3 ± 0.1	−0.01 ± 0.02

The results are expressed as the mean ± standard error in mg 100 mL^−1^. Values represented with different letters in a column are statistically different at *p* ≤ 0.05. † significant factor in multifactor analysis. ^‡^ not significant factor in multifactor analysis. TPC—total phenolic content; HCA—total hydroxycinnamic acids; TFL—total flavonoids; CAR—total carotenoid content.

**Table 8:** Changes in the bioactive compounds and nutritive index during storage for pasteurized smoothies.

Variable	n	TPC	HCA	TFL	Vitamin C	CAR	Nutritive Index
**Storage**		*p* ≤ 0.01 ^†^	*p* ≤ 0.01 ^†^	*p* ≤ 0.01 ^†^	*p* ≤ 0.01 ^†^	*p* ≤ 0.01 ^†^	*p* ≤ 0.01 ^†^
0 days	8	944.2 ± 10.3 ^a^	44.3 ± 2.5 ^a^	66.7 ± 0.7 ^a^	5.6 ± 0.0 ^a^	15.0 ± 0.5 ^a^	0.6 ± 0.1 ^a^
7 days	8	881.2 ± 10.3 ^b^	44.1 ± 2.5 ^a^	55.3 ± 0.7 ^b^	2.4 ± 0.0 ^b^	13.1 ± 0.5 ^b^	0.0 ± 0.1 ^b^
14 days	8	819.9 ± 10.3 ^c^	33.9 ± 2.5 ^b^	55.6 ± 0.7 ^b^	0.0 ± 0.0 ^c^	11.3 ± 0.5 ^c^	−1.0 ± 0.1 ^c^
21 days	8	786.6 ± 10.3 ^c^	24.8 ± 2.5 ^c^	55.2 ± 0.7 ^b^	0.0 ± 0.0 ^c^	10.5 ± 0.5 ^c^	−1.7 ± 0.1 ^d^
**SAMPLE MEAN**	32	858.0 ± 5.1	36.8 ± 1.2	58.2 ± 0.3	2.0 ± 0.0	12.6 ± 0.2	−0.5 ± 0.1

The results are expressed as the mean ± standard error mg 100 mL^−1^. Values represented with different letters in a column are statistically different at *p* ≤ 0.05. ^†^ significant factor in multifactor analysis. TPC—total phenolic content; HCA—total hydroxycinnamic acids; TFL—total flavanols; CAR—total carotenoid content.

**Table 9:** Changes in the colorimetric values for the smoothie samples under HP during storage.

Variable	n	L*	a*	b*	C*	H*	ΔΕ
**Pressure**		*p* ≤ 0.01 ^†^	*p* ≤ 0.01 ^†^	*p* ≤ 0.01 ^†^	*p* ≤ 0.01 ^†^	*p* ≤ 0.01 ^†^	*p* ≤ 0.01 ^†^
350 MPa	16	46.4 ± 0.0 ^a^	15.0 ± 0.0 ^a^	19.1 ± 0.0 ^a^	24.3 ± 0.0 ^a^	51.8 ± 0.0 ^a^	2.8 ± 0.0 ^b^
450 MPa	16	43.5 ± 0.0 ^b^	14.2 ± 0.0 ^b^	16.1 ± 0.0 ^b^	21.5 ± 0.0 ^b^	48.6 ± 0.0 ^b^	3.4 ± 0.0 ^a^
**Time**		*p* ≤ 0.01 ^†^	*p* ≤ 0.01 ^†^	*p* ≤ 0.01 ^†^	*p* ≤ 0.01 ^†^	*p* ≤ 0.01 ^†^	*p* ≤ 0.01 ^†^
5 min	16	45.4 ± 0.0 ^a^	14.6 ± 0.0 ^a^	17.9 ± 0.0 ^a^	23.1 ± 0.0 ^a^	50.5 ± 0.0 ^a^	3.6 ± 0.0 ^a^
15 min	16	44.6 ± 0.0 ^b^	14.6 ± 0.0 ^b^	17.3 ± 0.0 ^b^	22.7 ± 0.0 ^b^	49.9 ± 0.0 ^b^	2.7 ± 0.0 ^b^
**Storage**		*p* ≤ 0.01 ^†^	*p* ≤ 0.01 ^†^	*p* ≤ 0.01 ^†^	*p* ≤ 0.01 ^†^	*p* ≤ 0.01 ^†^	*p* ≤ 0.01 ^†^
0 days	8	44.7 ± 0.0 ^d^	15.4 ± 0.0 ^a^	17.5 ± 0.0 ^b^	23.3 ± 0.0 ^b^	48.6 ± 0.0 ^d^	2.7 ± 0.0 ^d^
7 days	8	45.7 ± 0.0 ^a^	14.9 ± 0.0 ^b^	18.0 ± 0.0 ^a^	23.4 ± 0.0 ^a^	50.3 ± 0.0 ^c^	3.36 ± 0.0 ^a^
14 days	8	44.8 ± 0.0 ^c^	14.1 ± 0.0 ^c^	17.4 ± 0.0 ^d^	22.4 ± 0.0 ^c^	50.9 ± 0.0 ^b^	3.12 ± 0.0 ^c^
21 days	8	44.9 ± 0.0 ^b^	14.1 ± 0.0 ^d^	17.4 ± 0.0 ^c^	22.4 ± 0.0 ^c^	51.0 ± 0.0 ^a^	3.31 ± 0.0 ^b^
MEAN	32	45.0 ± 0.0	14.6 ± 0.0	17.6 ± 0.0	22.9 ± 0.0	50.2 ± 0.0	3.12 ± 0.0

The results are expressed as the mean ± standard error. Values represented with different letters in a column are statistically different at *p* ≤ 0.05. ^†^ significant factor in multifactor analysis.

**Table 10:** Changes in the colorimetric values for the smoothie samples under P during storage.

Variable	n	L*	a*	b*	C*	H*	ΔΕ
**Storage**		*p* ≤ 0.01 ^†^	*p* ≤ 0.01 ^†^	*p* ≤ 0.01 ^†^	*p* ≤ 0.01 ^†^	*p* ≤ 0.01 ^†^	*p* ≤ 0.01 ^†^
0 days	2	47.3 ± 0.02 ^a^	13.95 ± 0.0 ^c^	19.03 ± 0.0 ^c^	23.59 ± 0.0 ^c^	53.76 ± 0.0 ^a^	3.77 ± 0.0 ^b^
7 days	2	47.1 ± 0.02 ^b^	13.93 ± 0.0 ^c^	17.37 ± 0.0 ^d^	22.26 ± 0.0 ^d^	51.28 ± 0.0 ^b^	3.45 ± 0.0 ^c^
14 days	2	26.7 ± 0.02 ^c^	21.51 ± 0.0 ^a^	30.83 ± 0.0 ^a^	37.59 ± 0.0 ^a^	21.05 ± 0.0 ^c^	23.04 ± 0.0 ^a^
21 days	2	26.47 ± 0.0 ^d^	21.29 ± 0.0 ^b^	30.67 ± 0.0 ^b^	37.34 ± 0.0 ^b^	21.05 ± 0.0 ^c^	23.07 ± 0.0 ^a^
**MEAN**	8	36.88 ± 0.0	17.67 ± 0.0	24.47 ± 0.0	30.19 ± 0.0	36.78 ± 0.0	13.33 ± 0.0

The results are expressed as mean ± standard error. Values represented with different letters in a column are statistically different at *p* ≤ 0.05. ^†^ significant factor in multifactor analysis.

**Table 11:** Microbiological counts (CFU/mL) of the high-pressure, pasteurized, or untreated (control) samples stored at 4 °C.

Microorganism Type	Treatment	Day of Storage			
		0	7	14	21
Aerobic mesophilic bacteria	Control	5.3 × 10^4^ *			
	HP	n.d.	n.d.	n.d.	n.d.
	Pasteurization	n.d.	n.d.	n.d.	n.d.
*Enterobacteriaceae*	Control	1.6 × 10^2^ **			
	HP	n.d.	n.d.	n.d.	n.d.
	Pasteurization	n.d.	n.d.	n.d.	n.d.
*L. monocytogenes*	Control	n.d.			
	HP	n.d.	n.d.	n.d.	n.d.
	Pasteurization	n.d.	n.d.	n.d.	n.d.
*Salmonella* sp.	Control	n.d.			
	HP	n.d.	n.d.	n.d.	n.d.
	Pasteurization	n.d.	n.d.	n.d.	n.d.
Yeasts and molds	Control	4 × 10^5^ ***			
	HP	n.d.	n.d.	n.d.	n.d.
	Pasteurization	n.d.	n.d.	n.d.	n.d.

* n.d.—not detected * not satisfactory criterion (≤10^4^ CFU/mL); ** not satisfactory criterion (≤10^2^ CFU/mL); *** not satisfactory criterion (≤10^5^ CFU/mL).
